# Can Plant Materials Be Valuable in the Treatment of Periodontal Diseases? Practical Review

**DOI:** 10.3390/pharmaceutics13122185

**Published:** 2021-12-17

**Authors:** Anna Gościniak, Magdalena Paczkowska-Walendowska, Agnieszka Skotnicka, Marek A. Ruchała, Judyta Cielecka-Piontek

**Affiliations:** 1 Department of Pharmacognosy, Faculty of Pharmacy, Poznań University of Medical Sciences, Rokietnicka 3, 60-806 Poznań, Poland; anna.gosciniak@student.ump.edu.pl (A.G.); mpaczkowska@ump.edu.pl (M.P.-W.); 2 Department of Pharmaceutical Technology, Faculty of Pharmacy, Poznan University of Medical Sciences, Grunwaldzka 6, 60-780 Poznań, Poland; askotnic@ump.edu.pl; 3 Department of Conservative Dentistry and Endodontics, Poznan University of Medical Sciences, Bukowska 70, 60-812 Poznań, Poland; maruchala@ump.edu.pl

**Keywords:** periodontitis, herbal drugs, plant extracts, oromucosal route

## Abstract

Periodontal diseases are one of the most significant challenges in dental health. It is estimated that only a few percent of the worldwide population have entirely healthy teeth, and according to WHO, oral diseases may affect up to 3.5 billion people worldwide. One of the most serious oral diseases is periodontitis, an inflammatory disease affecting periodontal tissues, caused by pathogenic bacteria and environmental factors such as the ageing population, abuse of tobacco products, and lack of adequate oral hygiene due low public awareness. Plant materials are widely and successfully used in the management of many conditions, including periodontitis. Plant materials for periodontitis exhibit antibacterial, anti-inflammatory, antioxidant activities and affect the periodontium structure. Numerous studies demonstrate the advantages of phytotherapy for periodontitis relief and indicate the usefulness of Baikal skullcap root, Pomegranate fruit peel and root cortex, Tea leaves, Chamomile flowers, Magnolia bark, Blackberry leaves and fruits, Cranberry fruits and Lippia sidoides essential oil. This review aims to analyze the use and applicability of selected plant materials in periodontitis management since it is of paramount importance to evaluate the evidence of the traditionally used plant materials in light of continuously growing interest in phytotherapy and its adjuvant role in the treatment of periodontitis.

## 1. Introduction

Poor oral health and its harmful consequences confer a substantial burden worldwide on people, healthcare systems and societies. The demographic shift and population ageing have significant implications for public health, mainly by increasing the prevalence of chronic diseases and disabilities [1,2]. As the burden of chronic diseases increases, healthcare systems’ priorities focus on the effective management of chronic conditions and the continuous optimization of the elderly population’s functioning [1]. Despite the global focus on chronic health problems of an ageing population, still, there is an inadequate emphasis on oral health [3]. According to The Global Burden of Disease 2010 Study, oral health problems accounted for 15 million disability-adjusted life years, implying an average health loss of 224 years per 100,000 population [4]. According to World Health Organization (WHO), oral diseases constitute a significant problem for numerous countries and their healthcare systems. Oral health conditions cause pain, discomfort, lead to a substantial decrease in the quality of life, and also determine growing care needs in later life [5]. It is estimated that oral diseases may affect even up to 3.5 billion people worldwide [2]. The most common oral conditions are tooth decay, periodontitis, loss of dentition, cancers of the lips and mouth [6], and dry mouth. Untreated infections can lead to oral cancers, one of the three most commonly diagnosed malignancies in the Asia Pacific, and in Europe, with the incidence ranging from 5 to 10 cases per 100,000 people [7]. WHO statistics on oral diseases present the continuous increase in the prevalence of oral health deterioration in both children and adults. Over 530 million children globally suffer from decay in primary teeth, approximately 60–90% of schoolchildren have experienced caries, with the disease being most prevalent in Asian and Latin American countries [8]. Despite the natural ageing process, common factors leading to tooth loss in adults are caries and severe periodontal diseases [5]. About 50% of Europeans suffer from at least one type of periodontitis, and in the 60–65 age group, the prevalence increases up to 70–85% [9]. About 30% of Europeans aged between 65 and 74 years no longer have permanent teeth, what significantly affects their functioning at physiological and neuromuscular levels as well as in psychological and social aspects of wellbeing [10,11] and consequently, their quality of life [12].

## 2. Periodontitis

Periodontitis is an infectious tissue disease with an inflammatory response. Periodontitis is characterized by microbially-associated, host-mediated inflammation resulting in periodontal attachment loss [13]. Periodontal tissues include the structures surrounding the tooth such as the gum, the mucosa of the alveolar process, alveolar bone, periodontium and root cementum. The periodontium supports the tooth, protects against oral microflora, and makes the attachment to the bone possible. The inflammatory process leads to the loss of connective tissue and alveolar bone, resulting in bone support loss and pathological tooth mobility [14].

There are three types of periodontitis [13]:

1. Necrotizing periodontitis
  - Necrotizing gingivitis
  - Necrotizing periodontitis
  - Necrotizing stomatitis
2. Periodontitis as a manifestation of Systemic Disease
3. Periodontitis.

The more recent classification differentiates periodontitis also into aggressive and chronic forms [13]. 

Initially the disease is asymptomatic. The early symptoms may exhibit as gingivitis, later progressing to periodontitis. Patients may notice gum bleeding, soreness, redness and swelling of the gums, during tooth brushing and when chewing the food [15]. The disease may become chronic with the increase in the amount of plaque. Initially, the cavities are mild [14]. The symptoms presenting later are abscess in the gingival tissue, unpleasant taste and smell in the mouth. Along with the disease progression, the alveolar and periodontium bones are resorbed and, as a consequence, gingival recession leads to loosening and ultimately the loss of the tooth [16,17].

### Etiopathogenesis of Periodontitis

The exact mechanism of periodontal pathogenesis is not fully understood. Still, the onset and progression of the disease are invariably associated with the imbalance in the periodontal and microbial homeostasis [18]. In periodontitis, the microorganisms colonizing the oral cavity dominate bacteria called a red-complex. Red-complex is formed by gram-negative pathological anaerobic bacteria such as *Porphyromonas gingivalis*, *Tannerella forsythia*, *Treponema denticola* [19,20]. It is believed that the presence of the red-complex bacteria is responsible for weakening the innate periodontal defence functions, stimulating untoward interaction between the host bacteria and dental plaque leading to tissue damage [21,22]. 

**Figure 1:**
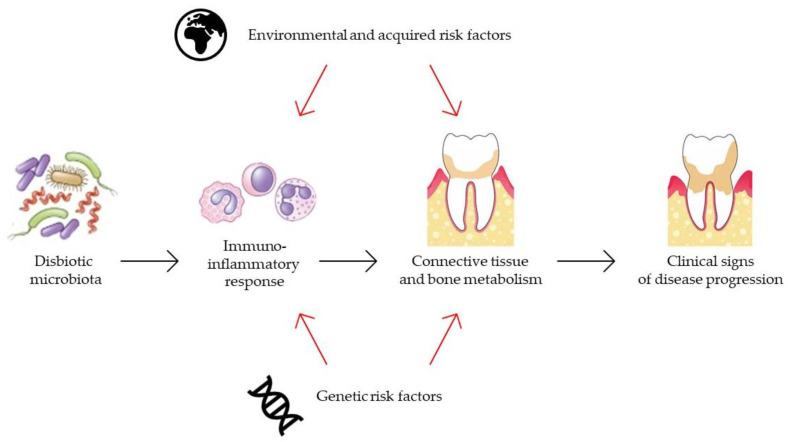
Model of pathogenesis of periodontitis.

The immune inflammation of tissues, gingivitis, and periodontitis, is a response to the chronic presence of plaque bacteria producing damaging factors such as chemotoxins, endotoxins and enzymes. The immune reaction aims to eliminate the damaging factors following the release of pro-inflammatory cytokines and other mediators of inflammation. However, prolonged exposure to pathogens suppresses this compensative process converting it to harmful, leading to a significant tissue damage. Therefore, the progression of periodontitis is the amalgamation of external factors such as pathogens and the host immune response, a direct cause of periodontal tissue damage. The long-term exposure to the factor causing the humoral response leads to the activation of osteoclasts, which begin the bone damage and activate the matrix metalloproteinase damaging the ligaments [17]. A graphical representation of the mechanism is shown in Figure 1.

Additionally, it has been noted that the oxidative stress caused by the antimicrobial reaction during periodontitis may be an additional cause of tissue damage. When inflammation occurs, the production of reactive oxygen species by the immune system (i.e., neutrophils and macrophages) increases dramatically [23]. They can directly cause tissue damage by lipid peroxidation, DNA damage, protein damage, and oxidation of essential enzymes, and in the meantime, act as signalling molecules or mediators of the inflammation process. In addition, reactive oxygen species can directly attack fibroblast and osteoblast cells and reduce collagen production in cells leading to periodontitis [24]. 

Understanding the pathogenesis of periodontitis is fundamental element for discovering effective targeted treatment. Currently, the preferred management strategies include using antioxidant agents, modifying the process of elimination of reactive oxygen species and supporting the bioprocesses protecting cells [25].

## 3. The Role of Herbs in the Treatment of Periodontitis

Plant materials are commonly and widely used to treat several medical conditions. Herbal products are often preferred over conventional drugs due to their complex biological activity, often favourable safety profile, lower therapy costs, biocompatibility, and low impact on the environment. Moreover, conventional, synthetic compounds usually cause more side effects, intolerance, and irresponsible use, for example, not evidence-based antibiotic therapy, leads to long-term global threat such as antibiotic resistance [26]. For example, chlorhexidine-based mouthwashes for periodontitis are associated with an unpleasant aftertaste, allergic reactions, tongue numbness discouraging use [27]. Concurrently, the interest in using herbal medicines is growing continuously to combat or prevent diseases, including periodontal diseases [28,29]. 

Due to the multifactorial aetiology and complex disease pathogenesis, the management of periodontitis is multidirectional [30]. The primary therapy goal is the reduction in bleeding and inhibition of the disease progression [31]. The ideal formulation should exhibit antibacterial, antioxidant, and anti-inflammatory properties to reduce dental plaque and limit the periodontal tissue damage caused by the progressive inflammatory process. The therapeutic outlook is the activity against the bacteria mentioned above, i.e., red-complex and the inhibition of plaque formation through the anti-adhesive effect. Reducing inflammation can be manifested by inhibiting both pro-inflammatory cytokines and proteinases such as collagenase [32]. The inhibition of the inflammatory processes minimizes the risk of periodontal tissues damage. The concept of advantageous effects in managing periodontitis includes the antioxidant effect of plant materials protecting tissues against the damaging effects of free radicals [33]. Bone resorption in the course of periodontitis is associated with the hyperactivity of RANKL (Receptor Activator for Nuclear Factor κB Ligand), a protein responsible for the maturation and activation of osteoclast cells [34]. The inhibition of bone resorption by the plant materials mentioned in the literature illustrates the purposefulness of their use.

Selected plant materials evaluated with proven effectiveness in the treatment and prevention of periodontitis are presented below.

### 3.1. Sage Leaves (Salvia officinalis)

*Salvia officinalis* is a shrub belonging to the *Lamiaceae* family. Active compounds of sage leaves are flavonoids and their glycosides, especially rosemary acid, ellagic acid, and luteolin-7-glucoside. The plant material contains the terpenes and terpenoids bornyl acetate, camphene, camphor, humulene limonene and ursolic acid [35]. Sage leaf is widely used in oral disorders for rinses or as one of the ingredients in complex preparations. One of the traditional uses stated in a monograph of the European Medicines Agency includes relief of mouth and throat inflammations (Table 1) [36]. Sage leaf has an anti-inflammatory effect helpful for the topical treatment of inflammatory diseases. Many studies have been carried out on compounds obtained from sage. Mansourabadi et al. [37] evidenced activity of flavanoid–salvigenin, Baricevic et al. [38] of ursolic acid—triterpenoid and Rodrigues et al. [39] of hydroalcoholic extract. Essential oils isolated from sage are a promising source of antibacterial agents. They may prevent oral infectious diseases, as confirmed in in vitro study by Tambur et al. [40]. However, the antibacterial activity of methanol extract against *Enterococcus faecalis* infection in root canals is lower than chlorhexidine activity. A randomized clinical trial aiming to evaluate the clinical effects of a mouthwash containing *Salvia officinalis* extracts on *S. mutans* (bacteria causing dental plaque) demonstrated the effectivness of sage mouthwash in a reduction in the count of *S. mutans* in dental plaque [41]. In contrast, the study by Mendes et al. [42] concluded that the extract has a moderate effect on periopathogenic bacteria and proved the activity against *S. mutans* in the combination of dichloromethane-soluble fraction with chlorhexidine only.

Extensive research on the progression of periodontitis has also been conducted. Anti-inflammatory effect of ethanolic extract of *Salvia sclarea* L., a plant belonging to the same genus as *Salvia officinalis* was investigated. Use of *S. sclarea* extracts significantly inhibits the inflammatory process by lowering IL-1β, IL-6 and TNF-α levels, reducing gingival tissue changes, and preserving alveolar bone resorption in lipopolysaccharide-induced periodontitis in rats [43]. A randomized, placebo-controlled, double-blind did not confirm the superior beneficial effect of the mouthwash with *S. officinalis* on inflammatory parameters and plaque indices compared with the placebo after 6 weeks [44]. On the other hand, *S. officinalis* gel has a potential anti-inflammatory role in the management of chronic periodontitis. The study by Aljuboori et al. [45] investigated both clinical and immunological parameters depending on the application of a gel containing sage extract preceded by scaling and root planning procedure and concluded a significant difference between the control and test group.

Combining sage with other raw materials with anti-periodontitis activity is also clinically relevant. Toothpaste with Swiss Medicinal Herbs (*Chamomilla recutita*, *Arnica montana*, *Echinacea purpurea*, and *Salvia officinalis*) diminished bacterial load specific for gingivitis/periodontitis in comparison to a control toothpaste [46]. preparations shown In Table 2 presented a variety of preparations containing sage leaves with proven efficacy in the management of periodontitis. 

### 3.2. Oak Bark (Quercus spp.)

Oak bark is a dried bark obtained from several oak species found in Europe—*Quercus robur*, *Quercus petraea*, *Quercus pubescens* belonging to a family of *Fagaceae.* Plant materials are rich in tannins and effectively treat periodontitis. The main active compounds found in oak bark are hydrolyzable and condensed (8–20%) tannins [47]. The European Pharmacopoeia provides the minimal tannins level reference value as 3% in the raw plant material of *Qercus* spp. expressed as pyrogallol and calculated per dried herbal material [48]. The phenolic acids—ellagic acid, gallic acid, protocatechuic acid, and vanillic acid are also present. The predominant compound is ellagic acid [47]. The bark of *Q. robur*, *Q. petraea*, *Q. pubescens* is on the official database the European Medicine Agency of pharmaco-therapeutic plants are presented in Table 1 [49] The traditional use in periodontitis described in the monograph targets symptomatic treatment of a low-level inflammation of the oral mucosa or skin [49]. Oak bark extract as an ingredient in dermatological medicines can be used to treat skin conditions [50]. Oak bark tannins have strong astringent, bactericidal, antioxidant, and anti-inflammatory properties [51,52]. Antibacterial properties of tannins from oak bark result from inhibition and deactivation of proteins through forming stable, insoluble complexes. The astringent effect prevents minor bleeding in the mouth associated with periodontitis progression. Studies conducted by Tsubanova et al. confirm the effectiveness of gel containing *Aloe vera* and oak bark extracts in periodontitis treatment [53,54,55]. The proven properties of oak bark extract permits the presumption that it could be an excellent adjunctive agent in periodontitis treatment (Table 2).

### 3.3. Peppermint Leaves (Mentha piperita)

Peppermint leaf is a plant raw material valued for its proven activity. *Mentha piperita* (*Lamiaceae*) is a cultivated natural hybrid of *Mentha aquatica* and *Mentha spicata.* In addition to the essential oil peppermint leaves typically contain 1.2–3.9% *(v/w)* of essential oil, various flavonoids such as luteolin and its 7-glycoside, rutin, hesperidin, and eriocitrin (eriodictyol 7-*O*-rutinoside). The main constituents of the monoterpenes fraction are menthol and menthone [56]. Other components include phenolic acids, e.g., rosemary acid [57]. The European Medicines Agency has published monographs on peppermint leaves and the essential oil extracted from peppermint (Table 1) [58]. It is well documented that the essential oil and extracts of *Mentha* species have antimicrobial, anti-inflammatory, and antioxidant properties [59,60,61,62] with antimicrobial activity against pathogens involved in the development of periodontal disease [63]. Peppermint oil or peppermint leaf extract are often present in oral preparations for their antiseptic properties and flavour correction value. Essential oils of *Mentha piperita* showed good antibacterial and antibiofilm activities in the study conducted by Karicheri et al. [64] concerning *Aggregatibacter actinomycetemcomitans*, a strain identified in periodontopathogenesis. Peppermint oil is also used in novel formulations, for example as an anti-ulcer and anti-inflammatory agent in self-emulsifying drug delivery system for the improvement of meloxicam’s solubility used in periodontal disease [65]. Furthermore, the study by Phaechamud et al. [66] successfully used peppermint oil to modify the release of doxycycline from the in situ formed gel systems with Eudragit RS. Above studies demonstrate a modern trend on the development of innovative drug forms by using plant-based raw materials.

### 3.4. Calamus Rhizome (Acorus calamus)

*Acorus calamus*, also known as a sweet flag, cradle root or muskrat, is a plant belonging to *Acoraceae*, common in central Asia and eastern Europe [67]. A total of fifty-three volatile organic compounds were isolated and identified. The main bioactive compound is acorenone followed by isocalamendiol whose content varies according to variety and region [68]. Monoterpene hydrocarbons, sequestrine ketones, α- and β-asarone, and eugenol were also identified [69]. Due to the toxicity of α- and β-asarone, the European Medicines Agency recommends that their concentration in herbal medicinal products should be kept to a minimum and that diploid variety lacking these compounds should always be preferred [70]. The traditional use by many Native American tribes utilized the calamus products as an anaesthetic for toothaches and to alleviate headaches [71]. Both calamus rhizome extract and essential oil can be used to manage periodontitis for their antibacterial, antioxidant, and anti-inflammatory properties. Antibacterial activity against peripathogenic bacteria such as *Actinomyces odontolyticus*, *Eikenella corrodens*, *Fusobacterium nucleatum*, although present, is weaker than with ethanolic extracts of sage or chamomile. The essential oil exhibits anti-inflammatory and antioxidant effects [72,73]. The methanol extract also shows antioxidant and antifungal activity, as demonstrated by Dinev et al. [74]. The active compound presented in calami rhizoma is asaroaldehyde [75], which according to Hwang et al. promotes osteogenic activity via the p38/extracellular-signal-regulated kinase signalling pathway. Thus, it may be used in the future as a compound of natural origin adjuvant in periodontal regeneration [76]. Additionally, anti-inflammatory properties of ursolic acid present in the calamus were confirmed by Baricevic et al. [38].

### 3.5. Baikal Skullcap Root (Scutellaria baicalensis)

*Scutellaria baicalensis,* also known as a Baikal skullcap or Chinese skullcap, and is a perennial herbaceous plant from the *Lamiaceae* (*Labiatae*) family. *Scutellaria baicalensis* is traditionally used in Chinese medicine as an anti-inflammatory agent [77]. Baicalin, a flavonoid with valued properties, is obtained from the root and is used to treat periodontitis. It can be used for treating periodontitis in oral gels and rinses for its antibacterial and antifungal properties. *S. baicalensis* has a strong antibacterial activity against oral pathogens, including *Streptococcus salivarius* or *Bacteroides gingivalis* [78]. A synergistic antibacterial effect has also been reported when used along with chlorhexidine, an agent known as the current gold standard for periodontitis [79]. Baicalin exhibits anti-inflammatory activity by inhibiting inflammatory process mediators such as arachidonic acid metabolites of nitric oxide [80,81]. In the animal periodontitis model, baicalin in 200 mg/kg dose regulates COX-2 expression in macrophages, plasma cells, fibroblasts, and gingival tissues [82]. 

Baicalin inhibits toll-like receptors expression and downstream signalling and mitigates inflammatory responses and the alveolar bone loss in experimental rat periodontitis [83]. *Scutellaria baicalensis* extract significantly inhibits alveolar bone resorption, reduces the production of pro-inflammatory cytokines expression in gingival tissues, and also improves the recovery of periodontal structures [84]. Baicalein attenuates the inflammatory response and restores osteogenesis in vitro in lipopolysaccharide-treated periodontal ligament cells, suggesting its potential use as a modulator of the host response in the treatment of periodontitis. [85]. Baicalin may inhibit metalloproteinase, e.g., collagenase activity, by more than 30%, which significantly moderates the destruction process of collagen in the gum tissue [86]. Baicalin has proven to protect the periodontal tissue in induced inflammation in the animal model [82,87]. Baicalin can significantly increase bone mineral density and height of the alveolar bone, and histological observations have shown that it may promote bone repair and regeneration [88]. Herbal toothpaste containing 0.5% Baikal skullcap extract and fluoride reduces plaque and biofilm viability more effectively than a conventional toothpaste containing fluoride only [89].

### 3.6. Pomegranate (Punica granatum)

*Punica granatum* L. known as pomegranate belongs to the family *Punicaceae*. As raw materials, fruit peel and root cortex are used. The main active compounds are flavonoids, ellagitannin, punicalagin, ellagic acid, vitamins and minerals, and alkaloids such as pelletierine [90,91]. Pomegranate extract exhibited anti-inflammatory activity through inhibition of NF-κB, decreasing NO level and PGE2 synthesis [92]. Pomegranate can potentially be used in the treatment of periodontitis due to its antioxidant activity. Pomegranate extract scavenges free radicals and reduces macrophage oxidative stress and lipid peroxidation [93]. Active compounds found in the raw material exhibit antibacterial action against gingivitis-causing microbes. Therefore, pomegranate mouthwash has an antiplaque effect and is effective against *Aggregatibacter actinomycetemcomitans*, *Porphyromonas gingivalis*, and *Prevotella intermedia* [94]. In addition, pomegranate glycolic extract has antimicrobial activity against *P. gingivalis* in vivo using *Galleria mellonella* [95]. Pomegranate juice is effective against dental plaque forming microorganisms decreasing the Colony Forming Units by 32% [96]. Pomegranate extract in gel form used in addition to mechanical cleaning exhibits the antigingivitis activity measured by Papillary Bleeding Index [97]. *Punica granatum* mouthwash showed similar improvement in bleeding and gingivitis score compared to chlorhexidine, with no impact on the reduction of plaque scores [98]. 5% *Punica granatum* peel extract decreases chronic gingivitis’ clinical signs and IL-1β levels without any adverse effect. *Punica granatum* chip may increase the effectiveness of conventional therapies and used in adjunction to scaling and root planning proves to be more effective than traditional management alone [99].

### 3.7. Tea Leaves (Camellia sinensis)

*Camelia sinensis*, belonging to the *Theaceae* family, is a plant with a proven healing potential mainly due to strong antioxidant activity. The main components of the leaves extract are catechins, including epigallocatechin gallate, epicatechin, epigallocatechin, and epicatechin gallate. Tea polyphenols inhibit the proliferation of *P. gingivalis*, and the minimum inhibitory concentration (MIC) for epigallocatechin gallate ranges from 250 µm/mL to 500 µm/mL pending on the strain tested. In addition, tea polyphenols at a concentration of 100 μg/mL reduced *P. gingivalis* adhesion on epithelial cells by 70% [100]. The catechins in green tea reduce the expression of peroxidation indicators and the pro-inflammatory cytokine index in rats after a topical application [101]. Green tea extract delays the disease progression of induced periodontitis in the animal model. The green tea application lowered as well the RANKL expression [102]. *Camelia sinensis* extract can reduce alveolar bone resorption by reducing cytokines and TRAP-positive multinucleated osteoclasts inhibiting periodontal inflammation [103]. Adjunctive topical extract therapy with a green tea gel reduces inflammation after 4 weeks of daily use [104]. In addition, a reduction of gingival inflammation and improvement in periodontal parameters was observed [105]. The clinical effect is also observed following oral supplementation of green tea. A significant improvement in clinical parameters and higher antioxidant capacity was observed [106]. Daily consumption of green tea has positively affected the probing depth and bleeding index [107]. The effectiveness against the plaque, gingivitis, oral hygiene, and salivary pH was higher following use of 0.5% tea mouthwashes compared to 0,2% chlorhexidine [108].

Traditionally used herbal medicinal products relieve fatigue and weakness (Table 1) [109].

### 3.8. Aloe vera Gel

Aloe is a plant belonging to the *Asphodelaceae* family. The following potentially active compounds are present in the leaves: vitamins soluble in water and lipids, complex or simple polysaccharides, especially acemannan—acetylated glucomannan, a mixture of polymers of various lengths, minerals, organic acids, and phenolic compounds [110,111]. Clinical study results proved antimicrobial activity against various periodontal pathogens—*Actinobacillus actinomycetemcomitans*, *Clostridium bacilli*, *Streptococcus mutans* and *Staphylococcus aureus*. The efficacy was similar to ofloxacin (5 mcg) and ciprofloxacin (30 mcg) [112]. The effectiveness of aloe vera extract was analysed in various formulations: toothpaste, mouthwash, and gel, including methods of obtaining the extracts. Bhat et al. [113] compared scaling and root planning with and without following of intra-pocket application of *Aloe vera* gel. The results confirmed that the oral application of gel improved patient’s oral condition. The gel seems to be more effective than scaling and root planning alone. Moghaddam et al. [114] run single-blind clinical trial confirmed that the adjunction to scaling improved clinical parameters such as plaque index, gingival index, and probing depth. The effectiveness of subgingivally applied *Aloe vera* gel was comparable to 1% ornidazole, 0.25% chlorhexidine, gluconate (Ornigreat™ gel) [115]. Likewise, the effect of aloe vera toothpaste on plaque index, the gingival index was similar to fluoride toothpaste alone [116], and its effectiveness was comparable to chlorhexidine [117]. Similarly, Penmetsa et al. has shown that *Ocimum sanctum* mouthwash*, Aloe vera* mouthwash, and chlorhexidine mouthwash are equally effective in reducing plaque, gingival, and bleeding 30 days apart. A study conducted by Karim et al. on 345 patients also showed that *Aloe vera* mouth rinse is equally effective in reducing periodontal indices as chlorhexidine. *Aloe vera* mouthwash was proven to be as effective on the 4-day de novo plaque formation as 0.2% chlorhexidine (the current gold standard) [118]. It improved bleeding index, plaque index, probing depth and clinical attachment level in patients with comorbidities such as type 2 diabetes mellitus and chronic periodontitis [119].

### 3.9. Chamomile Flowers (Matricaria chamomilla)

Chamomile flower, a representative of the *Asteraceae* family, is a plant material rich in essential oils, sesquiterpenes such as α-bisabolol and chamazulene, and flavonoid compounds. Traditionally, a herbal medicinal product is used to treat minor mouth and throat ulcerations and inflammations (Table 1) [120]. Chamomile flower extract is a complex of blends with topical anti-inflammatory and antioxidant properties [121]. The scope of common chamomile activity formed the basis for the use in periodontitis. Hans et al. [122] and Saderi et al. [123] confirmed the activity of essential oil against *P. gingivalis*. The essential oil inhibits the formation of a biofilm formed by *A. actinomycetemcomitans* and *T. denticola* bacteria. In Guimarães et al. study [124] dry extract of chamomile prevented inflammation and alveolar bone resorption in rats with ligature-induced periodontitis. The effectiveness of chamomile mouth rinses in reducing gingival bleeding was similar to 0.12% chlorhexidine solution [125]. Moreover, Agarwal et al. [126] showed that 1% chamomile extract improves periodontal patients’ clinical and microbiological presentation.

### 3.10. Magnolia Bark (Magnolia officinalis)

*Magnolia officinalis*, a plant from the *Magnoliaceae* family is a source of magnolol and honokiol—polyphenolic, compounds with anti-neurodegenerative, antioxidant, and anti-inflammatory activity [127]. Magnolia bark extract also has an advantageous impact on periodontitis management. Honokiol and magnolol have antimicrobial activity against *A. actinomycetemcomitans*, *P. gingivalis*, *P. intermedia*, *M. luteus*, and *B. subtilis* [128]. Magnolol may also be successfully used in the treatment of caries. The minimal inhibitory concentrations of magnolol, honokiol and chlorhexidine for *S. mutans,* a primary pathogen in caries, were 10, 10, and 0.25 µg/mL, respectively [129]. Walker et al. [130] demonstrated that magnolia bark extract inhibited the release of pro-inflammatory cytokines and the activity of metalloproteinases. Kim et al.’s in vitro study [131] proved that 75% ethanol magnolia cortex extract in the concentration of 60 μg/mL in 1% DMSO exhibited an anti-inflammatory effect on *P. gingivalis*-stimulated cells. Magnolol and honokiol exhibit anti-inflammatory and antioxidant activity [132,133]. In the animal model study, magnolol moderates and alleviates bone resorption and improves the structure of the periodontium [134]. In addition, clinical studies by Hellström et al. [135] confirmed the effectiveness of magnolia extract in reducing gingivitis when used for six-month in a dentifrice, in the concentration of 0.3%. 

### 3.11. Blackberry Leaves and Fruits (Rubus fruticosus)

Blackberries are one of the many representatives of the *Rosaceae* family. Due to the content of astringent tannins, blackberry leaves are used to manage oral cavity disorders. Blackberry leaf rinses have been used to treat pharyngitis, relieve pain and for mouth ulcers [136]. The raw material is effective in treating oral thrush. Chewing blackberry leaves strengthens the gums and may be used as a dressing to heal abscesses [137]. Leonti et al. [138] reported the use of blackberry leaves, imported from Italy for gingivitis and abscess treatment. The active compounds present in the raw material have anti-inflammatory properties. Epicatechin, ellagic acid and quercetin derivatives inhibit the secretion of pro-inflammatory cytokines [139,140,141]. In addition, hyperoside, high in content in the raw plant material, inhibits the activation of the nuclear factor-kappa B (NF-κB) signalling pathway, suggesting the potential application of blackberry leaves in the treatment of inflammatory diseases [142]. In addition to the leaves, blackberry fruits are also effective in the treatment of stomatitis. Active compounds present in blackberry fruit exhibit anti-inflammatory, antioxidant and antiviral activity [143,144,145]. Antibacterial activity against pathogenic periodontal pathogens and *S. mutans* has been noted by González et al. [146].

### 3.12. Cranberry Fruit (Vaccinum macrocarpon)

*Vaccinum macrocarpon* is a plant that belongs to the *Ericaceae* family. The main active compounds present in the cranberry fruit are proanthocyanidins—compounds with advantageous properties in periodontitis treatment. Proanthocyanidins found in cranberries mainly consist of epicatechin subunits with at least one A-type bond. The main properties are antiadhesive, antibacterial activities and inhibition of collagenase and proteinase activity. One of the common use of cranberry fruits is in the treatment of urinary tract infections (Table 1) [147] The likely mechanism of its advantageous effect is the inhibition of bacterial adhesion to the urinary tract wall. The anti-adhesive impact is also being investigated in dental diseases. The study of cranberry juice effect on *Streptococcus* bacteria biofilm formation confirmed significant inhibition of bacteria adhesion and, consequently, prevention of dental plaque formation [131]. The results of Weiss et al. study proved a reduction in *Streptococcus* count in saliva after 6 week-use of mouthwash containing extracts from cranberry fruit [132]. Cranberries have proven antibacterial activity against red-complex bacteria. The active components from cranberries inhibit biofilm formation [148]. In addition, products obtained from cranberry juice inhibit the proteinases of *P. gingivalis*, *T. forsythia* and *T. denticola* and type I collagen and transferrin degradation by *P. gingivalis* [149]. Moreover, Tipton et al. [150] suggested the role in the inhibition of NF-κB and MMP-3, through the regulation of fibroblast inflammatory response. A-type cranberry proanthocyanidins can interfere with osteoclastic cell maturation and physiology and thus prevent bone resorption [151]. Furthermore, the consumption of cranberry functional beverages improves gingival and plaque indexes with no risk of developing caries [152].

### 3.13. Lippia Sidoides

Lippia sidoides, also known as *Alecrim pimento* and Pepper-Rosmarin, is an aromatic and medicinal plant species of the family *Verbenaceae,* originally from South America. The aromatic species of Lippia are commonly used as brews and inhalations for allergic rhinitis and the treatment of vaginal, oral, and throat infections. Additionally, the plant has potential use in periodontitis treatment. The main constituents of the essential oil obtained from *Lippia* are thymol, carvacrol, and eugenol [153]. These compounds exhibit potent antimicrobial activity against the typical oral pathogens, i.e., *Streptococcus* and *Candida albicans* [154]. In a study conducted by Botelho et al. [155], a standard 0.12% chlorhexidine mouth rinse reduced plaque index, gingival bleeding, and the number of *S. mutans* colonies more efficiently than *Lippia sidoides* essential oil 1% mouth rinse but did not reach a statistical significance. Both types of mouthwashes were equally effective in reducing microbial plaque and gingival inflammation. A gel containing 10% *Lippia sidoides* essential oil significantly reduced plaque and gingivitis after three months of treatment, similarily to chlorhexidine mouthwash [156]. There was a significant difference in the gingival index after 21 days of treatment with 10% *Lippia sidoides* gel [157]. In an animal model study with stimulated periodontal disease, the effect of topical herbal gel from 0.5% *Lippia sidoides* and 5% *Myracrodruon urundeuva* was investigated. The results showed that topical herbal gel decreased myeloperoxidase activity and significantly inhibited tumour necrosis factor-alpha and interleukin-1beta concentration in gingival tissue. Alveolar bone loss and tissue lesion with histopathological changes were considerably reduced [158]. Botelho et al. investigated thymol nanogel (1.2 mg/g) containing *Lippia sidoides* in acute periodontitis in rats. The treatment with nanogel reduced both lesions and myeloperoxidase activity in gingival tissue [159].

**Table 1:** Traditionally-used pharmaceutical dosage forms and therapeutic indications of selected plant materials according to European Union herbal monographs.

Plant Material	Pharmaceutical Dosage Form	Therapeutic Indications	References
*Salvia officinalis* L., *folium*	- Comminuted herbal substance as herbal tea for oral use. - Comminuted herbal substance for infusion preparation for oromucosal or cutaneous use. - Herbal preparations in liquid and solid dosage forms for oral use. - Herbal preparations in liquid or semi-solid dosage forms for cutaneous use or oromucosal use.	- Traditional herbal medicinal product for relief of inflammations in the mouth or the throat. - Traditional herbal medicinal product for relief of minor skin inflammations.	[36]
*Quercus robur* L., *Quercus petraea* (Matt.) Liebl., *Quercus pubescens* Willd., *cortex*	- Comminuted herbal substance as herbal tea for oral use or as a decoction preparation for oromucosal or cutaneous use. - Herbal preparation in solid or liquid dosage forms for oral use.	- Traditional herbal medicinal product for symptomatic treatment of minor inflammation of the oral mucosa or skin.	[49]
*Mentha x piperita* L., *folium*	- Herbal substance and comminuted herbal substance a herbal tea for oral use. - Herbal preparations in solid or liquid dosage forms for oral use.	No traditional use in the oral cavity.	[58]
*Camellia sinensis* (L.) Kuntze, non fermentatum *folium*	- Herbal substance or comminuted herbal substance as a herbal tea for oral use. - Herbal preparations in solid dosage forms for oral use.	No traditional use in the oral cavity	[109]
*Matricaria recutita* L., *flos*	- Herbal substance or comminuted herbal substance as an herbal tea for oral use and inhalation. - Herbal preparations in liquid dosage forms for oral use. - Herbal substance or comminuted herbal substance for infusion preparation for oromucosal or cutaneous use. - Herbal preparations in liquid dosage forms for preparation of dilutions for oromucosal or cutaneous use.	- Traditional herbal medicinal product for the treatment of minor ulcers and inflammations of the mouth and throat.	[120]
*Vaccinium macrocarpon* Aiton, *fructus*	- Herbal preparations in liquid dosage forms for oral use.	No traditional use in the oral cavity.	[147]

## 4. Oromucosal Formulations in Periodontal Diseases

### 4.1. Oromucosal Route

Oral mucosa and mouth have been positioned as one of the most common drug delivery routes, designed for both systemic and local drug action targets. The oromucosal route may grant effective absorption at the site of administration (local administration with systemic drug action), or the action may be limited to the administration site. Within the oral mucosal cavity, delivery of drugs is classified into three categories: (1) sublingual delivery (systemic delivery of drugs through the mucosal membranes lining the floor of the mouth), (2) buccal delivery (drug administration through the mucosal membranes lining the cheeks—buccal mucosa) and (3) local delivery (drug delivery into the oral cavity). Oral mucosa is highly vascularised, permeable, so active substances are well and fast absorbed to directly enter the systemic circulation, bypassing the gastrointestinal tract and first-pass metabolism in the liver, which can be manifested by fast onset of action [160]. The systemic effect following administration depends on the physicochemical properties of active substances. The hydrophilic and lipophilic properties of a drug mainly influence the success of the transport and absorption [161]. The absorption of hydrophilic substances depends on the size of the molecule, and with increasing molecular size, its oromucosal absorption decreases. Furthermore, pH value plays an important role; depending on the acid dissociation constant (pKa) and the saliva’s pH value, altering the drug ionization may occur. 

The oromucosal route is convenient and non-invasive. This route encompasses both alternative to oral or parenteral and patient-oriented approaches. Currently, European Pharmacopoeia (Ph. Eur.) lists two main categories of preparations: oromucosal preparations and preparations for dental use. Drugs administered via the sublingual and buccal routes are traditionally formulated as solid dosage forms (e.g., tablets, wafers, films, inserts, and patches, mucoadhesive tablet), liquid dosage forms (e.g., sprays and drops), and semi-solid dosage forms (e.g., gels). Specifically for dental use, preparations are formulated as gargle (concentrate, powder, or tablet for gargle), gingival solution, gel or a paste, lozenge or pastille, mouth wash (or tablet or powder for mouth wash), dental paste, stick or cream [162].

### 4.2. Oromucosal Preparations and Formulations in Periodontitis

Systemic administration of drugs for the treatment and prevention of oral diseases leads to therapeutic concentrations at the site of infection, but for short periods, forcing repeated dosing and, most importantly, exposing the system to medicines. Local delivery of medicines has been investigated for the possibility of overcoming the limitations of conventional therapy. Oral local drug delivery systems (OLDDSs) have been used both as immediate and sustained release forms. Drug dosage systems (DDS) are considered important adjuvant therapy for treating and preventing oral diseases [163]. Both already marketed and novel OLDDSs are formulated on a biopolymers matrix (biodegradable and biocompatible), which does not disturb the periodontal tissue regeneration process. Amongst various natural polymers, chitosan, a deacetylated product of chitin is widely used in drug delivery devices. Drug carriers investigated in OLDDSs are liposomes, micelles, and other copolymer nanoparticles and dendrimers [163]. In terms of polymers investigated for biodegradable matrixes the potential agents are the natural polymers such as chitosan, cellulose, alginate, and synthetic polymers such as poly(ε-caprolactone) (PCL), poly(d,l-lactide) (PLA), poly-(d,l-lactide-co-glycolide) (PLGA), poly- (vinylpyrrolidone) (PVP) and poly(vinyl alcohol) (PVAL) [164]. Currently, various novel approaches have been investigated. A wide array of OLDDSs is being investigated as treatment options such as fibres, stripes, films, microparticulate systems.

Despite the achievement of effective concentration of the drug at the sites of microbial infection and periodontal pockets, the above systems have specific disadvantages, e.g., the application of fibres causes patient’s discomfort, and the removal of fibre various degrees of gingival redness. Microparticulate systems have poor retention of the system into the periodontal pocket [165]. A very promising OLDDSs are in situ forming gels containing alginates, hyaluronic acid, and gellan gum or gelrite, which by partial adhesion to the surrounding tissue resulting in a significant residence time of the system at the site of action, prolonged residence times in periodontal pockets and increased exposure to the drug [166].

One of the novel OLDDSs type is mucoadhesive tablets, which, due to mucoadhesive polymers, deposit at the site of application, consequently, extend the duration of action in the affected area [167]. This form can be used for accurate and local dosing of the drug, with a flexible and controlled dosing schedule, and what’s more, this formulation is easy to use, improving patient’s compliance [168]. Numerous literature data on delivering polyphenols, especially resveratrol by this route is available. It has been proven that by the use of mucoadhesive polymers, a controlled and sustained release of resveratrol at the affected site can be achieved [169,170], which in combination with antioxidant and antibacterial properties can be a potential alternative in the treatment of periodontal diseases.

Table 2 presents herbal preparations available in the European Union and registered as non-prescription medicines for gingivitis and periodontitis. The availability of these preparations and the fact that they are commonly used confirm the interest in using herbal medicines in the treatment of periodontitis. The use of novel preparations, such as the aforementioned polymers or new plant materials, is a drive for the development of innovative dosage forms, required for the ageing population affected by periodontitis.

**Table 2:** Selected preparations marketed as non-prescription medicines in the European Union [171].

Marketed Product	Active Ingredient(s)	Manufacturer	Pharmaceutical Form	Registered Indications
Aperisan^®^ 20%	Liquid extract of sage leaves (*Salvia officinalis* L.),	Dentinox^®^ Gesellschaft für pharmazeutische Präparate Lenk and Schuppan KG, Berlin, Germany	oral gel (topical)	The symptomatic treatment of oral inflammation
Argol Essenza Balsamica^®^	Menthol, melissa oil, cinnamon oil, clove oil (*Syzygium aromaticum* L.), lemon oil (*Citrus limon* L.), nutmeg oil (*Myristica fragrans*), thyme oil (*Thymus* spp.), coriander oil (*Coriandrum sativum* L.), peppermint oil (*Mentha piperita* L.),	Alba Thyment Sp. z o.o., Suchy Las, Poland	oral solution (topical)	For aphthous and inflamed gums
Baikadent^®^	A complex of flavones isolated from the root of Baikal Scullcap (*Scutellaria baicalensis*)	Herbapol Wrocław S.A., Wrocław, Poland	oral gel (topical)	In the complementary treatment of superficial and deep periodontopathies; in the prophylaxis of periodontal diseases; in chronic inflammatory conditions of the oral cavity mucosa (also in case of injuries caused by dentures)
Dentosept^®^	A complex liquid extract of camomile basket (*Matricaria recutita* L.), oak bark (*Quercus* spp.), sage leaf (*Salvia officinalis* L.), arnica herb (*Arnica* spp.), calamus rhizome (*Acorus calamus* L.), peppermint herb (*Mentha piperita* L.), thyme (*Thymus* spp.)	Phytopharm Klęka S.A., Klęka, Poland	oral solution (topical)	Anti-inflammatory, anti-bacterial, disinfectant and astringent, in inflammation of the oral and pharyngeal mucosa, gingivitis and stomatitis (including inflammation of the tongue); superficial periodontitis; bleeding gums; adjunctive in periodontosis
Dentosept A^®^	A complex liquid extract of Chamomile basket (*Matricaria recutita* L.), oak bark (*Quercus* spp.), sage leaf (*Salvia officinalis* L.), arnica herb (*Arnica* spp.), calamus rhizome (*Acorus calamus* L.), peppermint herb (*Mentha piperita* L.), thyme herb (*Thymus* spp.)	Phytopharm Klęka S.A., Klęka, Poland	oral solution (topical)	In the inflammations of oral cavity and gums, aphthae, mouth sores (after dentures), as an aid in periodontosis
Herbadent^®^	Benzocaine, Salicylic Acid, Herbal Liquid Extract for Herbadent	Herbai a.s., Prague, Czech Republic	oral topical solution	To massage the gums, especially in periodontitis, gingivitis and periodontitis
Kamistad^®^ Gel	Matricaria liquid extract (*Matricaria recutita* L.), lidocaine hydrochloridum monohydricum	STADA Arzneimittel AG, Berlin, Germany	oral topical gel	Traditionally used as a remedy for mild inflammation of the gums and oral mucosa
Kamistad Senzitiv^®^	Matricaria Liquid Extract (*Matricaria recutita* L.) lidocaine hydrochloridum monohydricum	STADA Arzneimittel AG, Berlin, Germany	oral topical gel	Kamistad Sensitive is indicated for the treatment of minor infections of the gums and oral mucosa in adults and adolescents above 12 years old
Kamillosan Konzentrat^®^	Chamomile flower extract (*Matricaria recutita* L.)	MEDA Pharma GmbH and Co. KG, Bad Homburg, Germany	oral topical solution	As an adjunctive treatment for moist compresses, rinses or washes for inflammatory skin and mucous membrane disorders, including the oral cavity and gums
Kamillosan^®^ Mund- und Rachenspray^®^	Chamomile flower extract (*Matricaria recutita* L.)	MEDA Pharma GmbH and Co. KG, Bad Homburg, Germany	oral spray	Kamillosan mouth and throat spray is used in inflammation of the throat (tonsillitis) in colds without fever, inflammation of the mucous membranes of the mouth and gums
Mucosit^®^	Extract from chamomile (*Matricaria recutita* L.), calendula (*Calendula officinalis* L.) coltsfoot leaf (*Tussilago farfara*), oak bark (*Quercus* spp.), sage leaf (*Salvia officinalis* L.), thyme herb (*Thymus* spp.)*;* essential oils (mint and chamomile), allantoin	Herbapol Poznań S.A., Poznań, Poland	oral topical gel	Topically as an astringent, anti-inflammatory and antimicrobial agent and as a local anaesthetic, accelerating granulation and wound healing. The drug for topical application on oral mucosa as a traditionally used supportive agent in the treatment of periodontal diseases and inflammatory conditions of the oral cavity
Septosan fix^®^	Peppermint herb (*Mentha piperita* L.), thyme (*Thymus* spp.), sage leaf (*Salvia officinalis* L.),	Pharmaceutical Works POLPHARMA S.A., Starogard Gdański, Poland	herbs for infusion in sachets	Traditionally used as a disinfectant in acute and chronic inflammations of the oral and pharyngeal mucosa, and mouth, throat and gum inflammation

## 5. Conclusions and Future Perspectives

The presented summary has confirmed that plant materials could be successfully used in the management of periodontitis. Generally, plant materials therapy has a favourable safety profile with fewer side effects compared to conventional agents such as chlorhexidine. It is crucial, especially in polypharmacy and chronic illnesses, when side effects may become a reason for therapy termination. The consequences of periodontitis involve tooth loss and related ailments as well as other conditions predisposing to their occurrence with a more severe course or a need for aggressive treatment. It is confirmed that the relationship between periodontitis and diabetes manifests in a more severe course of the disease and the more extensive destruction of periodontal tissues in diabetic patients and, at the same time, poorer glycemic control in diabetic patients with periodontal diseases [172]. Periodontitis also predisposes to the occurrence of cardiovascular diseases. A greater arterial stiffness, a biomarker of cardiovascular disease risk, is observed [173]. Moreover, the risk of cardiovascular and thrombotic stroke doubles in patients with periodontitis [174]. Therefore, it is crucial to introduce the prevention and effective and safe management of periodontitis from the initial developmental stages. 

So far, herbal ingredients are promising, treatment option adjuvant to conventional management such as scaling, leading to improved results and reduction of the aggressive therapies burden. Plant raw materials show promising activity in preventing periodontitis [175]; however, further research is essential to evaluate their effectiveness and long-term safety. The medicinal potential of plant products can be exploited by increasing their bioavailability, for example, through nanoencapsulation, which has been applied successfully for baicalin [176]. The use of nanotechnology is a promising current strategy for periodontitis treatment. Encapsulation of active compounds in nanoparticles enables sustained release at specific sites at the specified designed profile and improves absorption [177]. Due to the high potential of novel OLDDSs as effective active pharmaceutical ingredient delivery systems both in treatment and prevention of development of localized periodontitis or in areas that do not respond to the standard mechanical therapy, further research is required to investigate the effectiveness and applicability of formulations containing plant materials as well as to compare various novel systems. The potential use of polymers such as chitosan is worth noting due to their biocompatibility, biodegradability, and adhesion ability. Option of great interest and potential is a local delivery system obtained from biomaterials of plants origin. The current data confirm that using hydrogels as adjunctive therapy to improve periodontal healing shows promising results [178,179,180]. In addition to biocompatibility and bio-friendliness plant, biomaterials are usually associated with a lower cost, better safety profile, and solid evidence may be used to substitute conventional therapies or adjuvant, increasing their effectiveness.
